# Correction: Controlling contractile instabilities in the actomyosin cortex

**DOI:** 10.7554/eLife.30537

**Published:** 2017-07-19

**Authors:** Masatoshi Nishikawa, Sundar Ram Naganathan, Frank Jülicher, Stephan W Grill

Nishikawa M, Naganathan SR, Jülicher F, Grill SW. 2017. Controlling contractile instabilities in the actomyosin cortex. *eLife* **6**:e19595. doi: 10.7554/eLife.19595.

Published 24, January 2017

In Materials and Methods, we found a conflict and typo in strain information.

What was referred to in the materials and methods section as strain number SWG010 should be corrected to SWG003. This strain (SWG003) was first published in Naganathan et al Elife 2014.

SWG003: *nmy-2(cp8[nmy-2::GFP + unc-119(+)]) I; unc-119(ed3) III; gesIs002[Ppie-1::Lifeact::tagRFP-T::pie-1UTR + unc-119(+)]*, for imaging of GFP labelled NMY-2 (the images shown in Figure 1A and B and in Figure 4C).

What was referred to in the materials and methods section as strain number SWG011 should be corrected to SWG012.

SWG012: *nmy-2(ges6[nmy-2::tagRFP-T + unc-119(+)]) I; xsSi5[cb-UNC-119 (+) GFP:: ANI-1 (AH+PH)] II; unc-119(ed3) III*, for imaging of tagRFP-T labelled NMY-2 and GFP labelled AHPH for a probe of active RhoA in the cortex (the images shown in Figure 1F, in Figure 2H and in Figure 3A and B).

The article has been corrected accordingly.

